# Lynch syndrome-associated chordoma with high tumor mutational burden and significant response to immune checkpoint inhibitors

**DOI:** 10.1007/s10014-023-00461-w

**Published:** 2023-04-22

**Authors:** Naoki Shinojima, Kazutaka Ozono, Haruaki Yamamoto, Sakiko Abe, Rumi Sasaki, Yusuke Tomita, Azusa Kai, Ryosuke Mori, Takahiro Yamamoto, Ken Uekawa, Hirotaka Matsui, Kisato Nosaka, Hiroaki Matsuzaki, Yoshihiro Komohara, Yoshiki Mikami, Akitake Mukasa

**Affiliations:** 1 Department of Neurosurgery, Kumamoto University Hospital, 1-1-1 Honjo Chuo-Ku, Kumamoto, 860-8556 Japan; 2 Department of Diagnostic Pathology, Kumamoto University Hospital, Kumamoto, 860-8556 Japan; 3 Department of Neurosurgery, Saiseikai Kumamoto Hospital, Kumamoto, 861-4193 Japan; 4 Department of Cancer Genome Center, Kumamoto University Hospital, Kumamoto, 860-8556 Japan; 5 Department of Obstetrics and Gynecology, Kumamoto University Hospital, Kumamoto, 860-8556 Japan; 6 Department of Respiratory Medicine, Kumamoto University Hospital, Kumamoto, 860-8556 Japan; 7 Department of Molecular Laboratory Medicine, Graduate School of Medical Sciences, Kumamoto, University, Kumamoto, 860-8556 Japan; 8 Department of Cancer Treatment Center, Kumamoto University Hospital, Kumamoto, 860-8556 Japan; 9 Department of Hematology Rheumatology and Infectious Diseases, Kumamoto University Hospital, Kumamoto, 860-8556 Japan; 10 Department of Cell Pathology, Graduate School of Medical Sciences, Kumamoto University, Kumamoto, 860-8556 Japan

**Keywords:** Clival chordoma, Tumor mutational burden, Lynch syndrome, Immunotherapy, Immune-checkpoint inhibitor

## Abstract

Chordoma is a rare malignant bone tumor arising from notochordal tissue. Conventional treatments, such as radical resection and high-dose irradiation, frequently fail to control the tumor, resulting in recurrence and re-growth. In this study, genetic analysis of the tumor in a 72-year-old male patient with refractory conventional chordoma of the skull base revealed a high tumor mutational burden (TMB) and mutations in the *MSH6* and *MLH1* genes, which are found in Lynch syndrome. The patient and his family had a dense cancer history, and subsequent germline genetic testing revealed Lynch syndrome. This is the first report of a chordoma that has been genetically proven to be Lynch syndrome. Chordomas usually have low TMB; however, this is an unusual case, because the TMB was high, and immune checkpoint inhibitors effectively controlled the tumor. This case provides a basis for determining the indications for immunotherapy of chordoma based on the genetic analysis. Therefore, further extensive genetic analysis in the future will help to stratify the treatment of chordoma.

## Introduction

Chordomas are rare malignant bone neoplasms arising from notochordal tissue, with approximately 40% and 60% occurring in the clivus and spine, respectively, mainly in the sacrococcygeal region [1]. Clival chordoma accounts for 0.5% of all primary brain tumors, with an incidence of approximately one per million per year [1, 2]. Additionally, it is a refractory tumor that is challenging to cure even after radical resection or high-dose irradiation, and the 5 year survival rate is 60–90% [2–5]. Histologically, there are conventional, dedifferentiated, and poorly differentiated chordomas. The median overall survival is approximately 100 months for conventional and 45–50 months for dedifferentiated or poorly differentiated chordoma, indicating that chordomas have different prognoses based on histology [6, 7].

Here, we present a case of a conventional clival chordoma that recurred and rapidly grew after gross total resection. After several high-dose radiotherapy sessions using Gamma Knife surgery, the chordoma proved difficult to control. Multigene panel testing for cancer revealed a rare high tumor mutational burden (TMB) chordoma. Treatment with an immune checkpoint inhibitor (ICI) was initiated, and it effectively controlled the refractory chordoma. Additionally, we observed mutations in the *MSH6* and *MLH1* genes that, with the patient’s family history of cancer, led us to strongly suspect the presence of Lynch syndrome. Germline genetic testing followed, and Lynch syndrome was diagnosed. However, chordoma complications in patients with genetically confirmed Lynch syndrome have not been reported in the literature. Here, we report for the first time a case of refractory clival chordoma with high TMB in a patient with Lynch syndrome treated effectively with an ICI.

## Case presentation

### Clinical summary

**Fig. 1:**
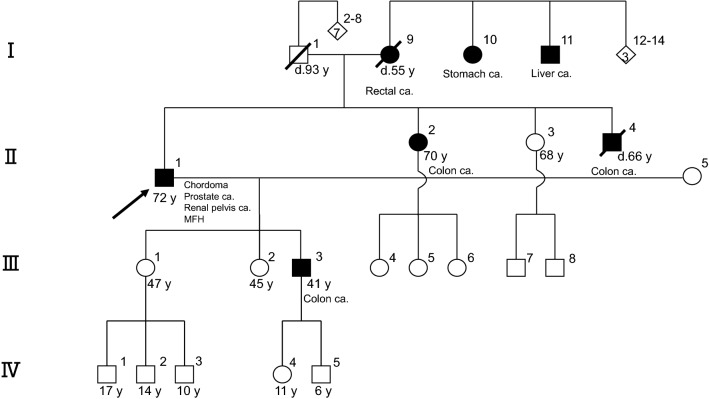
Patient’s pedigree showing a family history of cancer and the patient’s history of multiple cancers. The arrow indicates the presenting patient. Filled circles or squares indicate individuals who developed cancer. *Ca.* cancer, *MFH* malignant fibrous histiocytoma, *y* year-old, *d.* dead

A 72-year-old male patient presented to a local hospital after experiencing diplopia for 1 month. He had had a malignant fibrous histiocytoma of the right thigh and prostate cancer when he was in his 40 s and 60 s, respectively. He also had a significant family history of cancer: his son, sister, and brother had colon cancer, his mother had rectal cancer, his maternal aunt had stomach cancer, and his maternal uncle had liver cancer (Fig. 1).

**Fig. 2:**
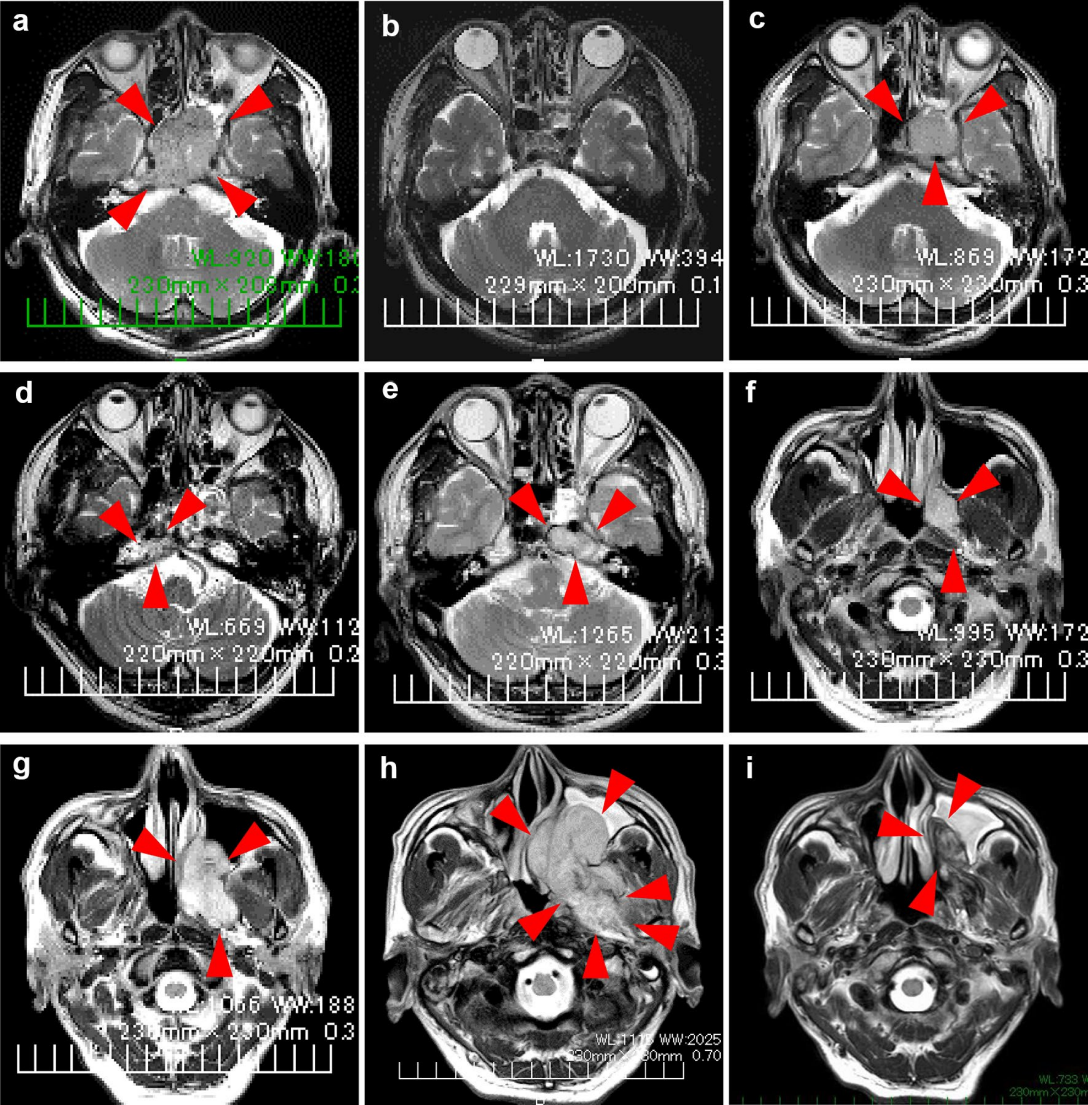
T2-weighted magnetic resonance images of the tumor at different stages of its development. **a** preoperatively; **b** 1 month postoperatively; **c** at first recurrence, 4 months postoperatively; **d** at second recurrence, 3 months after irradiation. The tumor has recurred outside the irradiated area; **e** 9 months after the first irradiation. The tumor has extended into the left cavernous sinus; **f** the tumor extends toward the maxillary sinus 16 months after initial irradiation; **g** 18 months after the first irradiation, the tumor continues to grow; **h** after two doses of an immune checkpoint inhibitor (pembrolizumab), the tumor is still growing; and **i** after the third dose of pembrolizumab, the tumor begins to shrink. It continues to shrink for the remainder of the course of treatment (seven doses in total)

Magnetic resonance imaging revealed a tumor extending from the clivus into the sphenoid sinus. The preoperative diagnosis was clival chordoma (Fig. 2a), and gross total resection was performed using an endoscopic endonasal transsphenoidal approach (Fig. 2b). The histopathological diagnosis was conventional chordoma. However, 4 months postoperatively, the patient experienced diplopia and left facial pain. A 3 cm in diameter recurrent lesion was observed (Fig. 2c), and the lesion shrank after the patient underwent Gamma Knife irradiation. However, 3 months after irradiation, recurrence was observed outside the irradiated area (Fig. 2d), and the patient was re-irradiated. Nine months after the first irradiation, the tumor recurred in the left cavernous sinus (Fig. 2e), and the patient was irradiated for a third time. However, the tumor continued to grow, extending toward the maxillary sinus (Fig. 2f). Subsequent genetic analysis of the chordoma revealed a high-TMB tumor, and ICI pembrolizumab, a humanized anti-programmed cell death protein 1 (PD-1) monoclonal antibody, was indicated. Conversely, before treatment could be initiated, the patient had hematuria and cancer of the renal pelvis. The recurrent chordoma continued growing, and his symptoms, including left facial pain, worsened. 22 months postoperatively (18 months after the first irradiation; Fig. 2g), the first pembrolizumab dose was administered, and the patient underwent a right nephrectomy for cancer of the renal pelvis, and the histopathological diagnosis was papillary urothelial carcinoma. The tumor grew during the period that the first two doses of pembrolizumab were administered (Fig. 2h). However, after the third dose, the tumors began to shrink. Notably, the patient’s symptoms improved considerably after the seventh dose of pembrolizumab (Fig. 2i). The patient continued to receive treatment with pembrolizumab; his left facial pain is currently considerably reduced, and the left abducens palsy tends to improve.

### Pathological findings

**Fig. 3:**
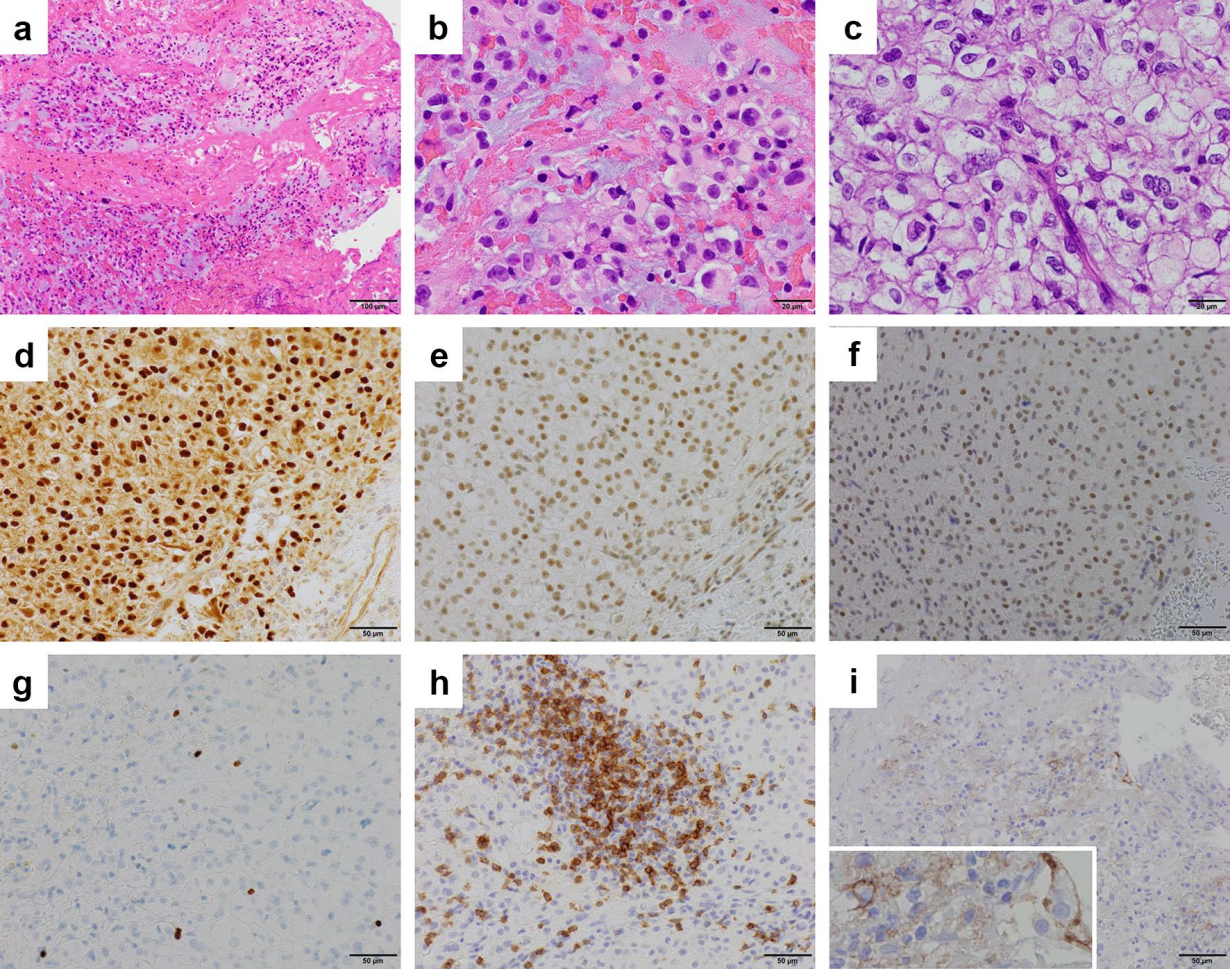
Histopathology. H&E staining at **a** 100x, **b** 400x, and **c** 400x. Findings of the immunohistochemical staining (all 200x) for **d** brachyury, **e** INI1, **f** BRG1, **g** Ki-67, **h** PD-1, and **i** PD-L1. Typical histological features of conventional chordoma with abundant extracellular matrix **a**, **b** the area is composed of cohesive sheets of larger epithelioid cells with relatively severe nuclear atypia and pleomorphism. No myxoid stroma is present (**c**). Diffuse nuclear staining for brachyury (**d**). Intact INI1 (**e**) and BRG1 (**f**) expression. PD-1-positive intratumoral lymphocytes (**h**). PD-L1-positive tumor cells (**i**). *H&E* hematoxylin and eosin; *PD-L1* programmed death-ligand 1

The pathological diagnosis was performed according to the World Health Organization Classification of Tumors, Soft Tissue, and Bone Tumors (5th ed.). The tumor comprised epithelioid cells with lightly eosinophilic cytoplasm, forming short chords, nests, and single cells within the extracellular myxoid matrix. Physaliphorous cells with vacuolated bubbly cytoplasm were also present. Larger epithelioid cells with clear to light eosinophilic cytoplasm were arranged in a solid sheet-like pattern, with minimal to a completely absent extra myxoid matrix. Nuclear atypia and pleomorphism were heterogeneous throughout the neoplasm, ranging from mild to relatively severe. Multinucleated cells with bizarre nuclei were occasionally observed. There were two mitotic figures/10 HPF (field of view diameter: 0.55 mm), both on hematoxylin and eosin- and phospho-histone H3-stained section. The Ki-67 labeling index was 2–3%. Necrosis was not evident. Immunohistochemically, tumor cells were positive for brachyury. There was no complete loss of INI1 (SMARCB1) or BRG1 (SMARCA4) expression. Based on these findings, this tumor was diagnosed as a conventional chordoma (Fig. 3). Interestingly, multiple scattered foci of lymphocytes in the intratumoral region were present. Most intratumoral lymphocytes were positive for PD-1. Approximately 1–2% of tumor cells showed programmed death-1 (PD-L1) partial or complete membrane staining at weak to moderate intensity. PD-L1 displayed weak cytoplasmic staining only in < 1% of immune cells, including lymphocytes.

The primary antibodies used were as follows: INI1 (SMARCB1) (clone: 3E10, abnova, Taipei, Taiwan) at dilution of 1:200, BRG1 (SMARCA4) (clone: EPR 3912, Abcam, Cambridge, UK) at dilution of 1:100, PD-1 (clone: NAT105, abcam, Cambridge, UK) at dilution of 1:50, and PD-L1 (clone SP142, Spring Bioscience, Pleasanton, CA, USA) at dilution of 1:500.

### Genetic analysis of the chordoma

Genome profiling using FoundationOne® CDx (Foundation Medicine, Inc., Cambridge, MA) was performed using genomic DNA extracted from formalin-fixed paraffin-embedded tissue of the tumor specimen. The results were equivocal regarding microsatellite instability; nonetheless, the TMB was high at 10 mutations per megabase (Muts/Mb), and mutations were also identified in the *APC* (L616fs*18)*, CD79B* (Y196H)*, INPP4B* (splice site 2375-2A > G)*, JAK1* (K860fs*16)*, MSH6* (F1088fs*5)*, and MLH1* (S698fs*5) genes. The genome panel test revealed mutations in the following two DNA mismatch repair (MMR) genes observed in Lynch syndrome: *MLH1* and *MSH6*.

### Diagnosis of Lynch syndrome

Germline genetic testing for *MLH1* and *MSH6* was undertaken using genomic DNA extracted from the patient’s blood sample, and a variant was identified in *MLH1*, c.2092_2093del (p.Ser698Argfs*5). No pathological variants were detected for *MSH6*, a somatic variant. This, together with the history of cancer in the patient and his family, as shown in Fig. 1, led to a diagnosis of Lynch syndrome.

## Discussion

Chordoma is a rare malignant bone tumor that is frequently difficult to completely remove via surgery, and it may recur despite high-dose radiation therapy [2–5]. Therefore, new treatment options other than the standard chordoma treatment are needed. Immunotherapies for chordoma, including ICIs, such as nivolumab and vaccine therapies targeting brachyury, have recently been tested in clinical trials, with promising results [8]. TMB is the total number of exonic Muts/Mb of tumor DNA and a predictive biomarker of response to ICIs. ICIs are expected to control high-TMB tumors effectively [9–11]. In general, chordomas have a low TMB [12, 13], and ICIs are unlikely to provide effective treatment for them. However, the chordoma, in this case, was a high-TMB tumor, and we decided to use pembrolizumab to treat the tumor to achieve tumor shrinkage and control. Migliorini et al. reported that immunotherapy was effective in three refractory chordoma cases [14]. Their study described two patients whose tumors shrank dramatically after receiving pembrolizumab or nivolumab treatment; however, no correlation was found between PD-L1 or PD-1 expression and response to ICI treatment. It has been reported that pembrolizumab is effective in non-small cell lung cancer when the PD-L1 tumor proportion score (TPS) is 1% or greater [15]. In our case, the PD-L1 TPS was 1–2%. It was suggested that pembrolizumab was effective in chordoma and non-small cell lung cancer if the TPS was ≥ 1%.

No studies have been published regarding chordoma complications in patients with genetically confirmed Lynch syndrome, although Weber W et al. reported a possible chordoma complication of Lynch syndrome, where it had not been genetically determined to be Lynch syndrome [16]. Lynch syndrome is an autosomal dominant cancer predisposition syndrome caused by a germline mutation in one of the following four MMR genes: *MLH1, MSH2, MSH6, or PMS2* [17, 18]. Standard Lynch syndrome diagnosis begins with reviewing the individual and family history of cancer and selecting high-risk patients based on the Amsterdam II criteria or the revised Bethesda guidelines [17, 18].

Whether or not this case is a Lynch syndrome-related tumor, one of the histological characteristics of carcinomas associated with Lynch syndrome, including medullary carcinoma of the colon and endometrial endometrioid carcinoma, is prominent intratumoral and peritumoral lymphocytes. Lymphocytic infiltration within the tumor was somewhat conspicuous in this case. Since intratumoral lymphocytes are not routinely examined in chordoma, it is impossible to determine whether this case is more lymphocyte-rich than usual chordomas. Commonly known genetic abnormalities in chordoma exclude the MMR gene mutation found in Lynch syndrome [12]. Moreover, one study has investigated the significance of the level of TMB in patients with Lynch syndrome; it concluded that TMB might be used to detect Lynch syndrome as a diagnostic biomarker in precision medicine [19]. This case revealed a tumor with genetic abnormalities not usually found in chordoma, namely, high-TMB and the MMR gene mutation found in Lynch syndrome.

In addition, this case was clinically refractory to high-dose irradiation and had a shorter recurrence period than the usual course of chordoma. Therefore, we considered this case to be a Lynch syndrome-associated tumor.

In this case, genomic panel testing of the tumor specimen revealed mutations in the *MLH1* and *MSH6* genes, and a history of cancer led to suspicion of Lynch syndrome, which was ultimately diagnosed by germline genetic testing. Thus, as the use of genetic tests, such as genome panel testing, increase, the diagnostic rate of familial tumors, including Lynch syndrome, may increase in the future. Familial tumors, such as the one in this case, are latent among refractory tumors; therefore, there may be latent cases, where treatment with ICIs may be effective. Accordingly, we believe that genome panel tests should be performed aggressively in patients with refractory tumors. Further widespread genetic analysis will stratify the treatment of chordoma.

In conclusion, this is the first case of high TMB refractory chordoma diagnosed as Lynch syndrome, where an ICI provided effective treatment.

## Data Availability

The data that support the findings of this study are available from the corresponding author, Naoki Shinojima, upon reasonable request.
